# Association between Oxidative Balance Score and Colorectal Cancer: Insights from NHANES 1999-2018

**DOI:** 10.7150/jca.126211

**Published:** 2026-04-08

**Authors:** Danjing Chen, Bingqin Xie, Hua Fang, Huajing Chang, Wenxin Qiu, Jiangwang Fang, Yunli Wu, Xian-E Peng

**Affiliations:** 1 Department of Epidemiology and Health Statistics, Fujian Provincial Key Laboratory of Environment Factors and Cancer, School of Public Health, Fujian Medical University, Fuzhou, 350108, China.; 2 Key Laboratory of Gastrointestinal Cancer (Fujian Medical University), Ministry of Education, School of Basic Medical Sciences, Fujian Medical University, Fuzhou 350108, China.

**Keywords:** colorectal cancer, oxidative stress, oxidative balance score, risk, cross-sectional study

## Abstract

**Background:**

Previous studies have indicated an association between high antioxidant exposure and colorectal cancer (CRC) risk in elderly populations. However, the relationship between the oxidative balance score (OBS) and CRC risk in the general population remains unclear.

**Objective:**

In this study, we used the OBS, which is based on dietary and lifestyle factors, to assess the oxidative stress (OS) status of individuals and to explore the association between OBS and CRC risk in American adults.

**Methods:**

Overall, 44,482 participants from the National Health and Nutrition Examination Survey (NHANES 1999-2018) were included in the cross-sectional study, of whom 340 had CRC. OBS, which consists of 20 dietary and lifestyle factors, was the exposure variable. Weighted multivariate logistic regression, subgroup analysis, and restricted cubic spline curves were used to assess the association between OBS and CRC. Sensitivity analyses were conducted to evaluate the robustness of the results.

**Results:**

Multiple logistic regression showed that the CRC risk decreased by 2.0% for each OBS unit added (*OR*= 0.98, 95%*CI*: 0.96-1.00, *P*=0.019). Compared with the lowest OBS reference group (T1), OBS in the highest tertile of OBS (T3) was associated with a reduced CRC risk (*OR*= 0.58, 95%*CI*: 0.39-0.84). Similarly, individuals in the highest dietary OBS tertile (T3) had a reduced CRC risk (*OR*= 0.63, 95%*CI*: 0.44-0.91), whereas no significant association was observed in lifestyle OBS and CRC.

**Conclusion:**

Higher OBS and dietary OBS were associated with CRC. A healthy lifestyle and antioxidant-rich diet may be useful for preventing CRC.

## Introduction

Colorectal cancer (CRC) is the third most common cancer worldwide and the second leading cause of cancer-related deaths^1^, ^2^. Although progress has been made in the treatment of CRC, improving overall survival, its incidence continues to increase^3^. It is estimated that there were more than 1.9 million new cases of CRC and 904,000 deaths in 2022, with a trend toward a younger population^1^, ^4^. Research indicates that CRC is ranked as the third leading cause of cancer-related deaths in U.S. adults younger than 50 years old^5^. Notably, CRC in the younger population is usually characterized by rapid progression and aggressiveness^6^, ^7^. As a result, CRC has become an increasingly serious health problem.

Oxidative stress (OS) is normally thought to be caused by an imbalance between the production and removal of reactive oxygen species (ROS) and plays an important role in the development of various cancers, including gastric, breast and colorectal cancers^8^-^11^. Several existing studies have found that higher levels of ROS can disrupt the function of biomolecules such as lipids, nucleic acids, and proteins, which can promote carcinogenesis^9^, ^12^. Sharifi-Rad et al.^13^ demonstrated that unhealthy dietary patterns and poor lifestyle habits may lead to the production of ROS, which can result in OS. Individual's oxidative balance is determined by the interaction of anti-oxidants and pro-oxidants. The oxidative balance score (OBS) is a way to measure exposure to anti-oxidants and pro-oxidants in diet and lifestyle, which reflect the overall balance between pro-oxidants and antioxidants^14^, ^15^. Researchers have combined multiple dietary and lifestyle exposures to estimate the OBS, which has been extensively used to assess the relationship between overall oxidative status and the risk of chronic diseases^14^. Recently, several epidemiologic studies have shown that a higher OBS is associated with a lower risk of chronic kidney disease, chronic obstructive pulmonary disease, non-alcoholic fatty liver disease, and other chronic diseases^16^-^18^.

Furthermore, the association between OBS and cancer risk has also drawn significant attention. Research indicates that higher OBS is associated with a reduced risk of developing breast cancer, ovarian cancer, cervical cancer, and other types of cancer^19^, ^20^. Chang Y et al. found that a higher dietary OBS was associated with a lower risk of gastrointestinal (GI) cancers (*OR*: 0.90, 95% *CI*: 0.76-0.99), indicating that oxidative stress imbalance may be involved in the development of GI cancers^21^. Currently, studies have explored the association between OBS and the risk of colorectal tumors. Several available case-control studies have found that higher OBS is associated with a reduced risk of colorectal adenomas^15^, ^22^-^24^. Meanwhile, researchers have explored the association between OBS and CRC in elderly populations. Dash C et al. found that higher antioxidant levels were negatively associated with CRC risk in people older than 50 years old^25^. In addition, recent studies have shown that older women with higher antioxidant exposure have a lower risk of CRC^26^, ^27^. However, existing research has primarily focused on elderly or specific gender populations. The relationship between OBS and CRC risk in the general population remains poorly understood, and further validation in larger, representative populations is warranted.

In this study, we analyzed a representative sample of adults from the United States (US), obtained from the National Health and Nutrition Examination Survey (NHANES) 1999-2018. We used the OBS^28^, which is based on 20 pro-oxidant and antioxidant factors, to assess the OS status of individuals and to explore the association between OBS and CRC.

## Methods

### Sources of data and study population

The data were extracted from NHANES 1999-2018, which are all available on the NHANES website (https://wwwn.cdc.gov/nchs/nhanes/Default.aspx). Of the 101,316 participants in NHANES 1999-2018, a total of 44,482 participants were included in the final analysis (**Supplementary Figure 1**). Exclusion criteria were as follows: a) missing information on cancer questionnaire; b) participants with cancers other than colorectal cancer; c) missing information on more than 4 out of the 20 components of the OBS; d) participants with missing weighting data.

The NHANES methods were authorized by the Ethics Review Board of the U.S. Centers for Disease Control and Prevention (CDC) National Center for Health Statistics, and all participants provided written informed consent. Therefore, analysis of the anonymized data did not require additional ethical review board approval 29. This study was conducted in accordance with the guidelines of the 1975 Declaration of Helsinki, as revised in 2013, and followed the Strengthening the Reporting of Observational Studies in Epidemiology (STROBE) guidelines for reporting of cross-sectional studies.

### Calculation of the oxidation balance score

OBS was calculated based on 16 dietary nutrients and 4 lifestyle factors that have been shown to influence oxidative stress^14^, ^30^. Dietary intake data were collected through 24-hour dietary recall interviews in NHANES, providing information on alcohol intake and 16 dietary nutrients such as fiber, carotenoids, riboflavin, niacin, total folate, vitamins B6, B12, C, and E, calcium, magnesium, zinc, copper, selenium, total fat, and iron. Lifestyle components considered included physical activity, body mass index (BMI), alcohol consumption, and smoking, with cotinine levels used to assess smoking. Physical activity was quantified by the total time spent walking, engaging in moderate-intensity and vigorous activity throughout the week. Pro-oxidant factors included total fat, iron consumption, smoking, alcohol intake, and obesity, whereas the remaining components were classified as antioxidants.

Each dietary component was categorized into three groups according to sex-specific tertiles. Antioxidants were scored from 0 to 2 and pro-oxidants from 2 to 0 (**Supplementary Table 1**). Participants with data available for at least 16 of the 20 OBS components were included in the study. For components with missing data, a score of 0 was assigned to the OBS. The total OBS, ranging from 0 to 40 in this study, was calculated by summing the scores across all 20 components, with higher values reflecting greater overall antioxidant exposure and lower oxidative stress potential.

### Identification of CRC cases

In the Medical Conditions section of the NHANES questionnaire, participants were asked the questions “Have you ever been told by a doctor or other health professional that you had cancer?” and “What kind of cancer?” Participants were defined as CRC cases if they reported a previous diagnosis of colon or rectal cancer. CRC cases were identified using the International Classification of Diseases for Oncology (ICD-O) codes for colon cancer (C18) and rectal cancer (C19-C20).

### Covariates

The study included confounding factors that may be associated with CRC, including age, sex, education, race, family income, hypertension, and diabetes. Education was grouped into three categories: less than high school, high school, or more than high school. Race was categorized as non-Hispanic White or others. Poverty income ratio (PIR) was classified into three categories: < 1.3, 1.3-3.5, or ≥ 3.5. Participants were classified as hypertensive if they had a self-reported history of hypertension, were currently taking antihypertensive medication, or had an average systolic blood pressure ≥ 140 mmHg or diastolic blood pressure ≥ 90 mmHg, based on at least three measurements. A self-reported history of diabetes or being on glucose-lowering medication or insulin were diagnosed as diabetes.

### Statistical analysis

Due to NHANES' complex sampling design, all analyses accounted for sample weights, strata, and clusters. For continuous and categorical variables, distributions were compared using the Mann-Whitney U and χ^2^ tests, respectively. These variables were then expressed as medians, interquartile ranges (IQR) and numbers (percentages), respectively. Odds ratios (*OR*) and 95% confidence intervals (95%*CI*) were calculated using survey-weighted binary logistic regression models to estimate the association between OBS and CRC. A total of three models were developed with stepwise inclusion of covariates: Model 1, which was unadjusted for any covariates; Model 2, adjusted for sociodemographic factors such as sex, age, education, race, and PIR; and Model 3, which further included adjustments for diabetes and hypertension. To better understand the relationship between OBS and CRC, OBS was separated into dietary and lifestyle components. Stratified analyses were conducted based on sex, age, education, race, PIR, hypertension, and diabetes to further investigate the relationship between OBS and CRC in various subgroups. An interaction test was conducted by including the OBS subgroup product term in a weighted multivariate logistic regression model to assess the difference between models with and without the interaction term. To examine the nonlinear relationship between OBS and CRC, restricted cubic spline (RCS) analysis was employed. Finally, a sensitivity analysis was conducted to assess the robustness of the results. The relationship between OBS and CRC was re-evaluated using weighted multivariate logistic regression, excluding participants with fewer than 19 OBS score items. Additionally, a 1:4 propensity score matching method was used to further assess the relationship between OBS and CRC.

Statistical analyses were performed using R software (version 4.3.3) and relevant R packages (“mice”, “survey”, “rms”, etc.). Statistical significance was set at a two-sided *P* value < 0.05.

## Results

### General characteristics of participants

A total of 44,482 participants from NHANES 1999-2018 were included in the cross-sectional study, of whom 340 had CRC. Participants with CRC were older, more likely to be non-Hispanic White and to have middle level of income. However, no significant differences were observed in the distribution of sex and education levels between the two groups (**Table 1**). The baseline characteristics comparison revealed that CRC patients had a higher prevalence of hypertension and diabetes. Compared with participants without CRC, participants with CRC showed significantly lower OBS, dietary OBS, and lifestyle OBS.

### Association between OBS and CRC

The weighted logistic regression analysis indicated a significant negative association between OBS and CRC (**Table 2**). After adjusting for age, sex, education, race, PIR, hypertension, and diabetes (Model 3), each 1-point increase in OBS was associated with 2.0% lower odds of CRC (*OR* = 0.98, 95%*CI*: 0.96-1.00, *P* = 0.019). Compared with the lowest tertile (T1), participants in the highest tertile (T3) were associated with lower odds of CRC (*OR* = 0.58, 95%*CI*: 0.39-0.84).

### Association between dietary OBS/lifestyle OBS and CRC

**Table 3** displays the association of dietary OBS and lifestyle OBS with CRC. In Model 3, compared with the lowest dietary OBS (T1), the highest tertile (T3) was associated with lower odds of CRC (*OR*= 0.63, 95%*CI*: 0.44-0.91). Meanwhile, dietary OBS was analyzed as a continuous variable. The results showed that every 1-point increase in dietary OBS was associated with 2.0% lower odds of CRC (P = 0.021). In contrast the lifestyle OBS showed no significant association with CRC, whether analyzed as categorical or continuous variable (*P* > 0.05). However, there was no multiplicative interaction between dietary and lifestyle OBS (*P* for interaction = 0.303).

### Strata analysis

**Figure 1:**
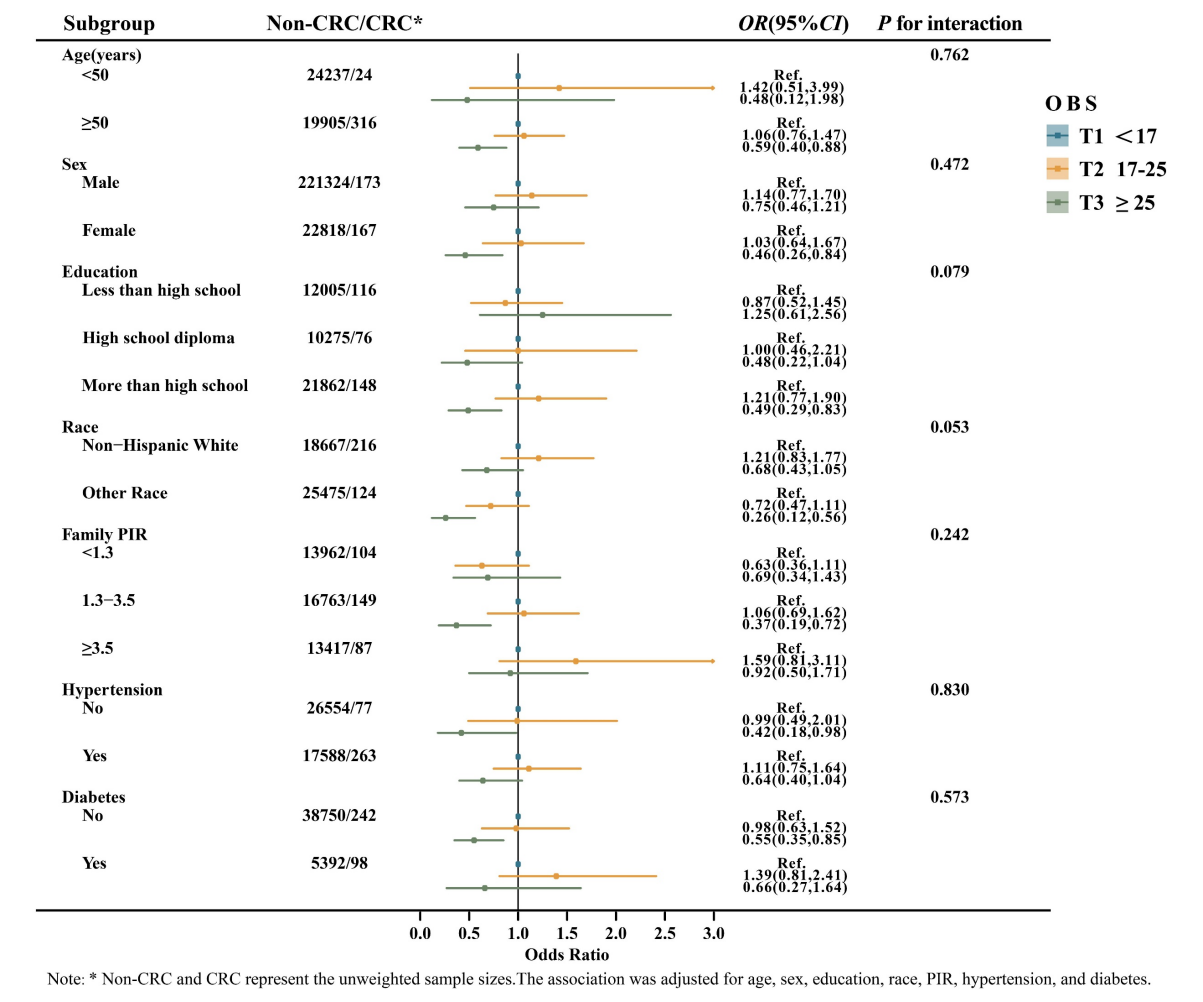
Subgroup analyses of the association between OBS and CRC.

OBS was analyzed as a categorical variable across subgroups defined by age, sex, education, race, PIR, hypertension, and diabetes status (**Figure 1**). The subgroup analysis revealed a significant relationship between OBS and CRC among individuals aged ≥ 50, female, with more than a high school education, of other races, PIR between 1.3-3.5, and those without hypertension or diabetes (*P* < 0.05). Nevertheless, a multiplicative interaction between each of the above variables and OBS was not observed (*P* for interaction > 0.05). In addition, dietary OBS was further stratified according to variables of interest (**Supplementary Figure 2**). The results suggested a consistent negative association between dietary OBS and CRC among individuals aged ≥ 50, female, other race, PIR between 1.3-3.5, and those without diabetes (*P* < 0.05). No multiplicative interaction was observed between these variables and dietary OBS (*P* for interaction > 0.05).

### RCS analysis

**Figure 2:**
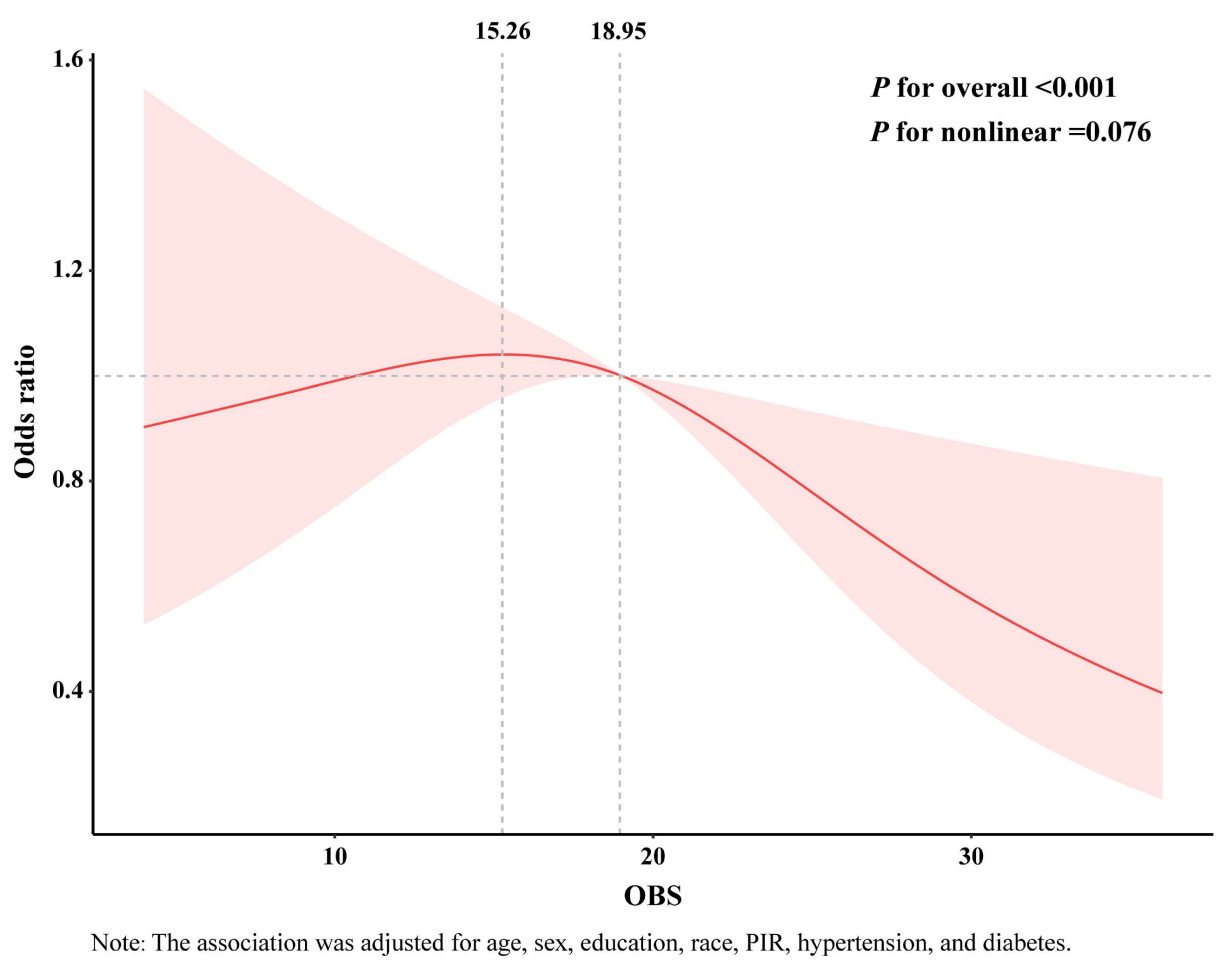
RCS analysis of the association between OBS and CRC. The association was adjusted for age, sex, education, race, hypertension, and diabetes.

RCS analysis revealed an inverted J-shaped relationship between OBS and CRC (*P* for nonlinear = 0.076) (**Figure 2**). When the OBS score exceeded 15.26, the curve showed a decreasing trend. Furthermore, when the score surpassed 18.95, higher OBS was associated with lower odds of CRC. Similarly, the RCS curves for the association of dietary OBS and CRC exhibited a comparable trend **Supplementary Figure 3**.

### Sensitivity analysis

We performed a sensitivity analysis to assess the robustness of the findings (**Table 4**). After excluding participants with fewer than 19 OBS components, weighted multivariable logistic regression analyses indicated that compared with the lowest tertile (T1) of OBS, the highest tertile (T3) of OBS was also associated with lower odds of CRC (*OR* = 0.64, 95%*CI*: 0.43-0.96). Additionally, a 1:4 propensity score matching approach was employed to reassess the association between OBS and CRC. The results consistently showed that individuals in the highest tertile (T3) of OBS had lower odds of CRC compared with those in the lowest tertile (T1) (*OR*= 0.64, 95%*CI*: 0.43-0.95). These analyses indicate that the inverse association between OBS and CRC remained consistent.

## Discussion

Results from this large-scale cross-sectional study using NHANES data indicated that higher categories of OBS and dietary OBS were strongly associated with lower odds of CRC when compared with the lowest categories. Additionally, the association between OBS and CRC was particularly evident among individuals aged ≥ 50 years, female, with more than a high school education, of other races, participants with a PIR of 1.3-3.5, and those without hypertension or diabetes. Overall, a higher OBS score was associated with lower odds of CRC.

An increase in OS is one of the main characteristics of cancer^31^. Excessive ROS/RNS production causes OS, which damages DNA, resulting in cell cycle arrest, transcription factor induction, replication errors, and genomic instability, all of which are associated with CRC^32^, ^33^. Meanwhile, population-based studies have shown that OS may be influenced by many modifiable lifestyle factors, including smoking, alcohol consumption, physical activity, and dietary factors 34, 35. Some foods and specific dietary components, such as red meat, fats, and refined carbohydrates, may act as potential pro-oxidants whereas vegetables, whole grains, tocopherols, carotenoids, and vitamin C may act as antioxidants 36-39. Lifestyle factors such as smoking and alcohol consumption will increase the production of reactive oxygen species^40^-^42^. Currently, more than 20 OBSs have been widely used in the assessment of individual OS^43^. OBS provides a convenient method a convenient way to assess pro-oxidant and antioxidant intake from combined diets and lifestyles, making it a promising tool.

In this study, an OBS score was constructed based on 16 dietary and 4 lifestyle indicators with regard to OS exposure to assess OS exposure and its association with CRC. The analysis revealed that a higher OBS was associated with lower odds of CRC, and this relationship remained consistent after controlling for potential confounders. Currently, the majority of evidence linking OS to CRC is derived from in vitro and animal studies, and the positive health effects of antioxidants on CRC are well documented^39^. In addition, an Iranian case-control study found that a negative correlation existed between total antioxidant capacity (TAC) and CRC risk (*OR*_Q3-Q1_= 0.25; 95%*CI*: 0.13-0.46)^44^. Another meta-analysis evaluated participants' oxidative stress status by derivatives of reactive oxygen metabolites (d-ROM) levels in blood samples, and showed that high oxidative stress was associated with an increased incidence of colorectal cancer (*HR*= 1.70; 95%*CI*: 1.15-2.51)^45^. A recent large prospective cohort study of the UK biobank (UKB) explored the association between OBS and CRC and has found that higher OBS is negatively associated with the development of CRC (*HR*_Q4-Q1_= 0.713; 95%*CI* 0.636-0.799) 46. This evidence further confirms our research findings.

Similar to previous studies^26^, ^27^, our study found that the association of OBS and dietary OBS with CRC was more pronounced (*P* < 0.05) in older individuals (≥ 50 years) and females. As with most cancers, CRC risk increases with age^47^. Additionally, cellular senescence negatively affects tissues and organisms. In the elderly individuals, dysregulated clearance processes and the accumulation of senescent cells lead to chronic inflammation and oxidative stress^48^, ^49^. These findings underscore the critical role of antioxidants in reducing CRC risk among the elderly population. Furthermore, estrogen exhibits antioxidant properties that enhance the body's defenses against oxidative stress^50^. However, during menopause, the decline in estrogen levels renders women more reliant on external antioxidants to counteract oxidative stress and mitigate CRC risk.

The major strengths of our study include investigating the relationship between OBS and CRC for the first time in a nationally representative survey. In addition, we employed the OBS proposed by Zhang W et al.^28^ based on NHANES dietary data, which may provide a more comprehensive assessment of overall oxidative stress compared with previous OBS methods^51^. Furthermore, this study considered multivariable confounding factors and employed cross-validation using continuous and multiple logistic regression models, thereby enhancing the reliability of the findings. Sensitivity analyses were also performed to further confirm the reliability of the results.

Nevertheless, this study has several limitations. First, due to its cross-sectional design, causal inference cannot be made, and reverse causation cannot be excluded, as CRC status may have influenced dietary and lifestyle habits. Second, the relatively small number of CRC cases may limit statistical power. Therefore, although our study observed a significant association between OBS, dietary OBS, and CRC, further validation in large prospective studies is warranted.

In summary, this cross-sectional study of U.S. adults suggests that a higher OBS is associated with lower odds of CRC. The incidence of CRC continues to rise and has shown a trend toward younger populations. Therefore, a healthy lifestyle and a diet rich in antioxidants may be beneficial for overall health, although causal effects on CRC cannot be established. However, further prospective studies are required to confirm these findings.

## Supplementary Material

Supplementary figures and tables.

## Figures and Tables

**Table 1:** General characteristics of the study subjects

	Overall N = 444821	Non-CRC N = 441421	CRC N = 3401	P-value2
**Age(years)**				**< 0.001**
< 50	24261 (60%)	24237 (60%)	24 (9%)	
≥ 50	20221 (40%)	19905 (40%)	316 (91%)	
**Sex**				0.300
Male	21497 (49%)	21324 (49%)	173 (45%)	
Female	22985 (51%)	22818 (51%)	167 (55%)	
**Education**				0.200
Less than high school	12121 (17%)	12005 (17%)	116 (22%)	
High school diploma	10351 (24%)	10275 (24%)	76 (22%)	
More than high school	22010 (58%)	21862 (58%)	148 (55%)	
**Race**				< 0.001
Other Hispanic	18883 (67%)	18667 (66%)	216 (82%)	
Other Race	25599 (33%)	25475 (34%)	124 (18%)	
**Family PIR**				0.047
< 1.3	14066 (23%)	13962 (23%)	104 (21%)	
1.3-3.5	16912 (36%)	16763 (36%)	149 (44%)	
≥ 3.5	13504 (41%)	13417 (41%)	87 (35%)	
**Hypertension**				< 0.001
**No**	26631(65%)	26554(65%)	77(25%)	
**Yes**	17851 (35%)	17588 (35%)	263 (75%)	
**Diabetes**				< 0.001
**No**	38992(91%)	38750(91%)	242(71%)	
**Yes**	5490 (9%)	5392 (9%)	98 (29%)	
**OBS**	20 (14, 26)	20 (14, 26)	18 (13, 22)	< 0.001
**Dietary OBS**	16 (10, 22)	16 (10, 22)	15 (9, 18)	< 0.001
**Lifestyle OBS**	4 (3, 5)	4.00 (3, 5)	3 (2, 5)	0.007

^1^N (unweighted) (%); Median (IQR) ^2^Chi-squared test with Rao & Scott's second-order correction; Wilcoxon rank-sum test for complex survey samples

**Table 2:** Odds ratio for the association between the OBS and CRC

OBS	Categorical models *OR* (95%*CI*)					Continuous models *OR* (95%*CI*)	
	T1	T2	T3	*P*_trend_		Per 1 point increase in score	*P* value
Model 1	Ref.	1.03(0.74,1.44)	0.46(0.32,0.66)	<0.001		0.97(0.95, 0.98)	<0.001
Model 2	Ref.	1.01(0.72,1.40)	0.49(0.33,0.72)	<0.001		0.97(0.95, 0.99)	0.001
Model 3	Ref.	1.09(0.79,1.50)	0.58(0.39,0.84)	0.005		0.98(0.96, 1.00)	0.019

Model 1: Adjusted with no covariates. Model 2: Adjusting for age, sex, education, race and PIR. Model 3: Additionally, adjusted for hypertension and diabetes.

**Table 3:** Odds ratio for the association between dietary OBS/lifestyle OBS and CRC

OBS	Categorical models *OR* (95%*CI*)					Continuous models *OR* (95%*CI*)	
	T1	T2	T3	*P*_trend_		Per 1 point increase in score	*P* value
Dietary OBS							
Model 1	Ref.	0.96 (0.68,1.34)	0.52 (0.37,0.73)	<0.001		0.97 (0.95, 0.99)	<0.001
Model 2	Ref.	0.95 (0.68,1.31)	0.56 (0.39,0.81)	0.002		0.97 (0.95, 0.99)	0.003
Model 3	Ref.	1.00 (0.73,1.38)	0.63 (0.44,0.91)	0.013		0.98 (0.96, 1.00)	0.021
Lifestyle OBS							
Model 1	Ref.	0.77 (0.58,1.03)	0.61 (0.38,0.99)	0.046		0.90 (0.84, 0.98)	0.009
Model 2	Ref.	0.77 (0.58,1.04)	0.66 (0.41,1.06)	0.083		0.91 (0.84, 0.99)	0.022
Model 3	Ref.	0.86 (0.66,1.21)	0.86 (0.52,1.41)	0.600		0.97 (0.89, 1.06)	0.500

Model 1: Adjusted with no covariates. Model 2: Adjusting for age, sex, education, race and PIR. Model 3: Additionally, adjusted for hypertension and diabetes.

**Table 4:** Odds ratio for the association between the OBS and CRC in sensitivity analysis

OBS	Exclusion of OBS score items less than 19			1:4 PSM		
	*OR* (95%*CI*)	*P* for trend^*^		*OR* (95%*CI*)	*P* for trend^*^	
T1	Ref.	0.030		Ref.	0.026	
T2	1.25 (0.90,1.74)			1.16 (0.79,1.70)		
T3	0.64 (0.43,0.96)			0.64 (0.43,0.95)		
						

^*^ Adjusted for age, sex, education, race, PIR, hypertension, and diabetes.

## Data Availability

Data could be publicly available from the website (https://www.cdc.gov/nchs/nhanes/index.htm, access on 10 March 2024).
