# Supplementary material for: Association between Oxidative Balance Score and Colorectal Cancer: Insights from NHANES 1999-2018
Source: J Cancer. 2026 Apr 8;17(4):890–7. doi: 10.7150/jca.126211 (PMC13105159; doi:10.7150/jca.126211)
Supplement: Supplementary file 1 — Supplementary figures and tables. [file jcav17p0890s1.pdf]

Association between Oxidative Balance Score and Colorectal Cancer: insights from NHANES 1999-2018

Danjing Chen

Supplementary Table 1 Components of the OBS

OBS components	Property	Male			Female		
		0	1	2	0	1	2
**Dietary OBS components**							
**Dietary fiber (g/d)**	A	<12.56	12.56-19.70	≥19.70	<10.10	10.10-16.31	≥16.31
**Carotene (RE/d)**	A	<98.83	98.83-306.29	≥306.29	<98.08	98.08-384.13	≥384.13
**Riboflavin (mg/d)**	A	<1.79	1.79-2.69	≥2.69	<1.34	1.34-2.02	≥2.02
**Niacin (mg/d)**	A	<20.65	20.65-29.75	≥29.75	<14.52	14.52-21.86	≥21.86
**VitaminB$_6$ (mg/d)**	A	<1.59	1.59-2.40	≥2.40	<1.13	1.13-1.77	≥1.77
**Total folate (mcg/d)**	A	<316.00	316.00-492.00	≥492.00	<251.00	251.00-388.96	≥388.96
**Vitamin B$_{12}$ (mcg/d)**	A	<3.36	3.36-6.20	≥6.20	<2.22	2.22-4.22	≥4.22
**Vitamin C (mg/d)**	A	<42.44	42.44-113.21	≥113.21	<38.01	38.01-98.49	≥98.49
**Vitamin E (mg/d)**	A	<5.82	5.54-9.42	≥9.42	<4.53	4.53-7.52	≥7.52
**Calcium (mg/d)**	A	<646.00	675.00-1072.00	≥1072.00	<499.24	499.24-849.00	≥849.00

**Magnesium (mg/d)**	A	<257.00	257.00-361.28	≥361.28	<187.00	187.00-283.43	≥283.43
**Zinc (mg/d)**	A	<9.75	9.75-15.10	≥15.10	<6.73	6.73-10.75	≥10.75
**Copper (mg/d)**	A	<1.12	1.12-1.57	≥1.57	<0.85	0.85-1.28	≥1.28
**Selenium (mcg/d)**	A	<94.94	94.94-141.80	≥141.80	<67.79	67.79-99.50	≥99.50
**Total fat (g/d)**	P	≥107.43	69.83-107.43	<69.83	≥75.79	50.98-75.79	<50.98
**Iron(mg/d)**	P	≥19.17	12.88-18.10	<19.17	≥14.32	9.65-14.32	<9.65
**Lifestyle OBS components**							
**Physical activity (LTPA)**	A	417.90	417.90-1136.80	≥1136.80	270.67	270.67-846.00	≥846.00
**Alcohol intake (g/d)**	P	≥30.00	0-30.00	0	≥15.00	0-15.00	0
**Body mass index (kg/m^2)**	P	≥29.17	25.55-29.17	<25.55	≥28.64	23.75-28.64	<23.75
**Cotinine (ng/mL)**	P	≥1.13	0.04-1.13	<0.04	≥0.17	0.04-0.17	＜0.04

A, antioxidant; ATE, alpha-tocopherol equivalent; OBS, oxidative balance score; P, pro-oxidant; RE, retinol equivalent.

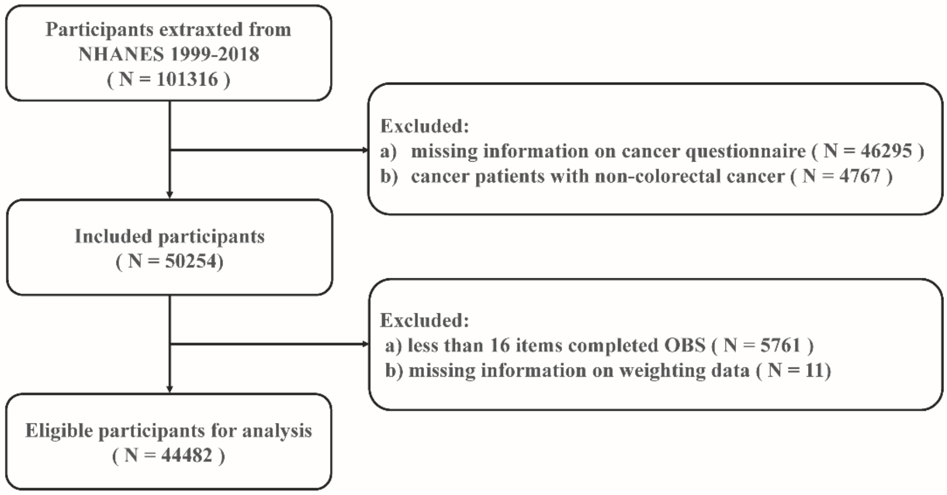

Supplementary Figure 1 Flowchart of patient inclusion from NHANES 1999 - 2018

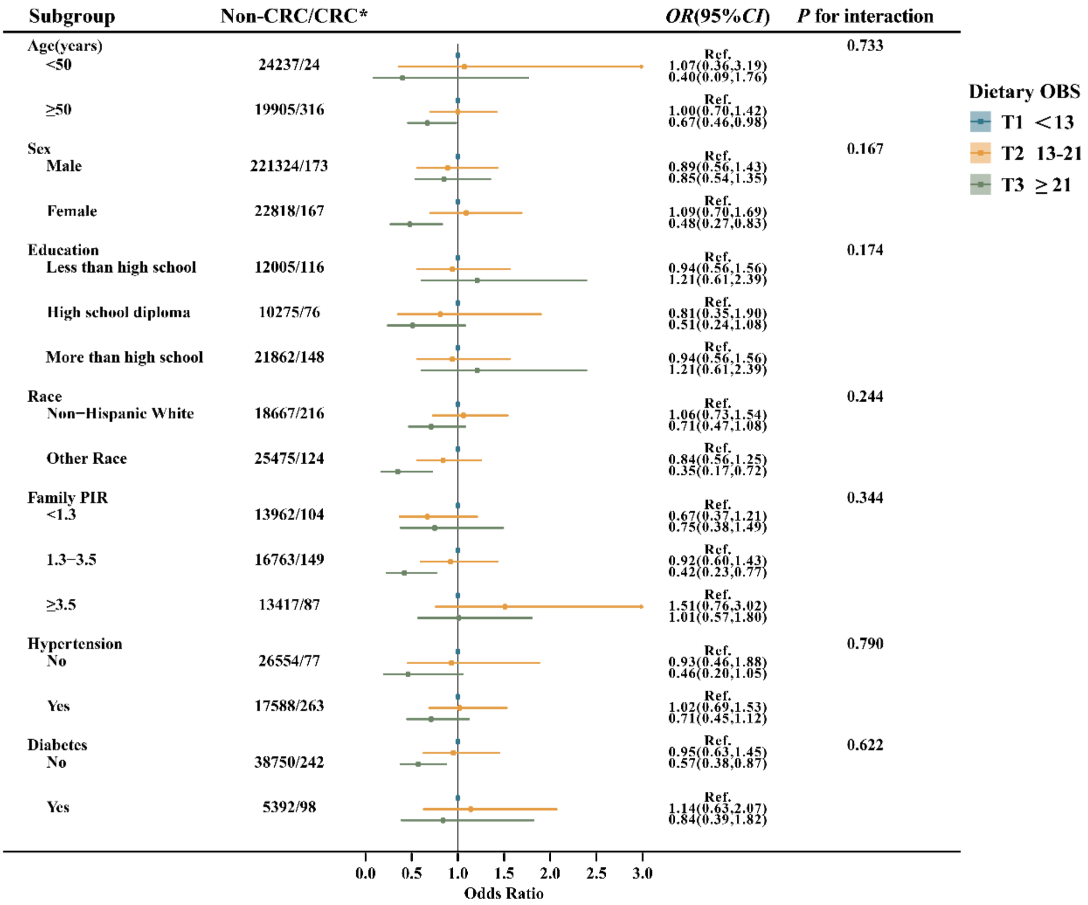

Note: * Non-CRC and CRC represent the unweighted sample sizes.The association was adjusted for age, sex, education, race, PIR, hypertension, and diabetes.

Supplementary Figure 2 Subgroup analyses of the association between dietary OBS and CRC

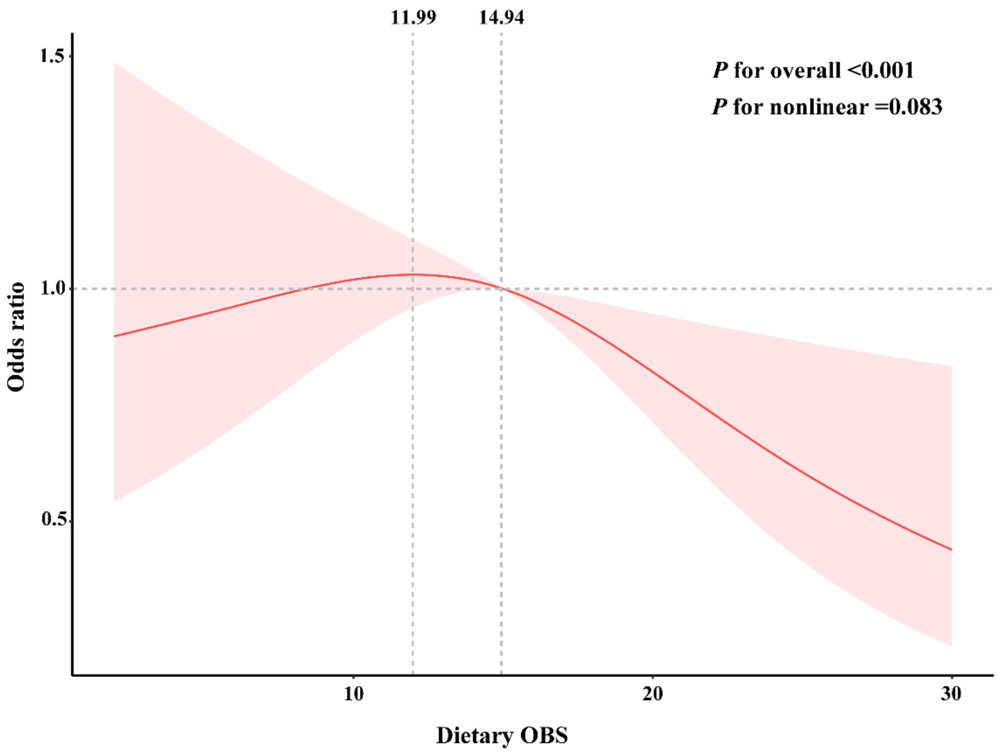

Note: The association was adjusted for age, sex, education, race, PIR, hypertension, and diabetes.

Supplementary Figure 3 RCS analysis of the association between Dietary OBS and CRC.
